# PIM1, CYP1B1, and HSPA2 Targeted by Quercetin Play Important Roles in Osteoarthritis Treatment by* Achyranthes bidentata*

**DOI:** 10.1155/2019/1205942

**Published:** 2019-07-15

**Authors:** Dujun Ma, Tian Yu, Liping Peng, Lixin Wang, Zhouwei Liao, Wenming Xu

**Affiliations:** Department of Orthopaedics, Shenzhen Traditional Chinese Medicine Hospital, Shenzhen, Guangdong 518033, China

## Abstract

**Aim:**

* Achyranthes bidentata *is one of the most commonly used Chinese herbal medicines (CHM) that is currently considered for the treatment of osteoarthritis. The purpose of this study was to reveal the mechanism of* Achyranthes bidentata in *osteoarthritis treatment based on the network pharmacology.

**Methods:**

The effective components of* Achyranthes bidentata* were firstly screened out from the TCMSP database with ADME property parameters. Then, osteoarthritis-related proteins targeted by the effective components were predicted based on the DrugBank and CTD databases. Subsequently, enrichment analysis and interaction network between targets of effective components and pathways were also studied. In addition, the differentially expressed genes (DEGs) of GSE55457 were used for validation of the osteoarthritis-related target proteins. Finally, the effective components–target molecular docking models were predicted.

**Results:**

A total of 10 effective components were identified, of which kaempferol and quercetin had 1 and 29 targets, respectively. There were 26 target proteins of quercetin related to the osteoarthritis. These targets were mainly enriched in mitochondrial ATP synthesis coupled proton transport, cellular response to estradiol stimulus, and nitric oxide biosynthetic process. In addition, there were three common proteins, PIM1, CYP1B1, and HSPA2 based on the DEGs of GSE55457, which were considered as the key targeted proteins of the quercetin.

**Conclusion:**

The docking of PIM1-quercetin, CYP1B1-quercetin, and HSPA2-quercetin may play important roles during the treatment of osteoarthritis by* Achyranthes bidentata.*

## 1. Introduction

Osteoarthritis, which is characterized by degeneration of articular cartilage and bone hyperplasia, is thought to be the most prevalent chronic joint disease [1, 2]. Multiple factors are known to affect the progression of osteoarthritis, containing joint instability, malalignment, obesity, aging population, muscle weakness, and peripheral neuropathy [2]. Pain and loss of function are the main clinical features, and the common treatments of osteoarthritis included nonpharmacological, pharmacological, and surgical approaches. At present, the common treatment strategies are mainly based on reducing pain, enhancing function, and delaying the operation. Nonsteroidal anti-inflammatory drugs and disease-modifying osteoarthritis drugs were usually used for therapeutic agents [3]. However, long-term application of these drugs will lead to side effects on the gastrointestinal tract [4].

Traditional Chinese Medicine (TCM) treatments, such as Chinese herbal medicine (CHM), acupuncture, and herbal patch, had been commonly used for osteoarthritis treatment in Asia for centuries [5]. Among TCM treatments, CHM was the most widely complementary and alternative medicine for enhancement of the symptoms of joints disease [6, 7]. However, due to the complex components in CHM, the application of them has been blocked by the absence of scientific understanding of treatment mechanism. It is urgent to reveal the mechanism of CHM in treating diseases.

*Achyranthes bidentata* is the root of* Achyranthes *(Amaranthaceae), which is widely distributed throughout China and other Asian countries. The* Achyranthes bidentata *is an important medicinal herb documented in Chinese Pharmacopeia.* Achyranthes bidentata* has the functions of nourishing the liver and kidney, strengthening the bones and bones, and removing blood stasis. It is a common medicine used by Traditional Chinese Medicine to treat the arthritis based on the kidney tonifying and blood activating method [8, 9].* Achyranthes bidentata* contains many types of compounds, including polysaccharides, saponins, anthrones, flavonoids, peptides, organic acids, and various trace elements, which play an important role in the treatment of osteoarthritis [10].

Quercetin is a polyhydroxy flavonoid that exhibits high nutritional and medicinal properties due to its diverse biological activities. Quercetin is widely found in the flowers, leaves, and fruits of plants. It is known that more than 100 kinds of herbs contain quercetin, such as Flos sophorae,* Chrysanthemum*, psyllium, and* Eleutherococcus* senticosus [11]. Mollica et al. reported that* Juglans regia* L. leaves which contained quercetin could alleviate the symptoms of diabetic rats [12]. Besides, the extracts of two species of broccoli form north-central Italy which also contained quercetin decreased the biochemical parameters in diabetic rats [13].

Network pharmacology aims to reveal the mechanisms of natural medicines at the system and molecular levels. At present, network pharmacology plays a significant role in the study of the mechanisms of various natural medicines and TCM, such as Liuwei Dihuang Pills [14], Qing-Luo-Yin [15], and essential oil from* Aegle marmelos* leaves [16]. In this study, a comprehensive pharmacology network was constructed based on several databases and bioinformatics methods to understand the pharmacological mechanism of* Achyranthes bidentata* on osteoarthritis.

## 2. Methods

### 2.1. Screening of Effective Components of Achyranthes bidentata

The components and other information of* Achyranthes bidentata* were obtained from the Traditional Chinese Medicine Systems Pharmacology Database and Analysis Platform (TCMSP, http://lsp.nwu.edu.cn/tcmsp.php), such as molecule name, molecular weight (MW), lipid/ water partition coefficient (A log P), hydrogen-bond donors/acceptors (Hdon/Hacc), oral bioavailability (OB), intestinal epithelial permeability (Caco-2), blood-brain barrier (BBB), drug-likeness (DL), and drug half-life (HL). TCMSP contains a large number of herbal entries, and the drug-target networks and drug-disease networks obtained from the TCMSP will help revealing the mechanisms of action of Chinese herbs and developing new herb-oriented drugs [17]. Based on the absorption, distribution, metabolism, and excretion (ADME) property parameters with OB≥40% and DL≥0.2, effective components of* Achyranthes bidentata* were obtained [17–19].

### 2.2. Prediction of Proteins Targeted by Effective Components

The targeted proteins of effective molecules were predicted based on the TCMSP and DrugBank (https://www.drugbank.ca/) databases by matching the UniProt ID of the UniProt database (https://www.uniprot.org/) to the gene symbol. The drug information provided by the DrugBank database is experimentally proven, clinically tested, or marketed, and the results are accurate and reliable [20].

### 2.3. Identification of Osteoarthritis-Related Proteins

Comparative Toxicogenomics Database (CTD, update 2019, http://ctdbase.org/) published by Mount Desert Island Biological Laboratory provides information on chemical-gene/protein interactions, chemistry-disease, and gene-disease relationships [21]. In order to identify the key proteins related to osteoarthritis among the targeted proteins above, CTD database was used with the “osteoarthritis” as the key word. Then, the proteins of the intersection of two sets were selected for further study.

### 2.4. Enrichment Analysis of Targeted Proteins

Gene Ontology (GO) analysis was performed by the ClueGO and CluePedia plug-ins of the Cytoscape software with adjusted P≤0.05 [22]. Kyoto Encyclopedia of Genes and Genomes (KEGG) pathway enrichment was carried out based on the clusterProfiler of R package with the P≤0.05. The kappa coefficient is used for consistency testing and can also be used to measure classification accuracy [23]. In the ClueGO plug-in, the kappa coefficient shows the relationship between GO terms based on overlapping genes. The GO functions were grouped based on kappa coefficients, and the higher the kappa coefficient, the stronger the term association strength.

### 2.5. Construction of Protein-Protein Interaction (PPI)

PPI analysis based on the target proteins was performed by the STRING database (Version: 10.0, http://www.string-db.org/) with a Required Confidence (combined score) > 0.4 [24]. After downloading the tsv format files, the PPI was constructed by the Cytoscape software [25].

### 2.6. Construction of Pharmacological Network

In order to more clearly display the regulation mechanism of effective components of* Achyranthes bidentata* on the osteoarthritis-related proteins, pharmacological network was constructed by Cytoscape software.

### 2.7. Verification of the Target Protein by Osteoarthritis mRNA Expressed Profiles

Osteoarthritis-related mRNA profiles of GSE55457 were used to verify the targeted proteins of effective components. From the GSE55457 data set, 10 samples from osteoarthritis patients (OA group) and 10 control knee cartilage samples (control group) were selected for further analysis. Briefly, raw data file was downloaded and read by affy package of R language software (Version 1.50.0, http://www.bioconductor.org/packages/release/bioc/html/affy.html). Then, data standardization preprocessing was performed by robust multi-array average (RMA) method, including background correction, standardization and normalization. Subsequently, the probes were annotated based on the platform annotation files. Unmatched probes were given up and the average value was considered as the expression level if the different probes matched the same gene. Finally, the differentially expressed genes (DEGs) between OA and control groups were identified by limma package (Version 3.26.9, http://bioconductor.org/packages/release/bioc/html/limma.html) with the P Value<0.05 and |log⁡2 Fold Change (FC)| > 0.585 [26].

### 2.8. Docking Prediction of Key Target Proteins and Effective Components

The intersection of target proteins of effective components and DEGs between OA and control groups was screened out for further study. The docking prediction of key target protein and effective components was carried out by the Protein Data Bank (PDB) database [27]. Briefly, the 3D structure of key target proteins was shown through PyMOL software (Version 2.1.1, https://pymol.org/dokuwiki/?id=). Besides, the experimental dissociation/inhibition constant value (pKd/pKi) of docking model was predicted by systemsDock online tool [28, 29]. The pKd/pKi represented binding affinity, and the higher the score, the stronger the affinity (1~10). In addition, the schematic diagram of protein-ligand interactions was drawn.

## 3. Results

### 3.1. The Overall Design of This Study and the Main Results

**Figure 1:**
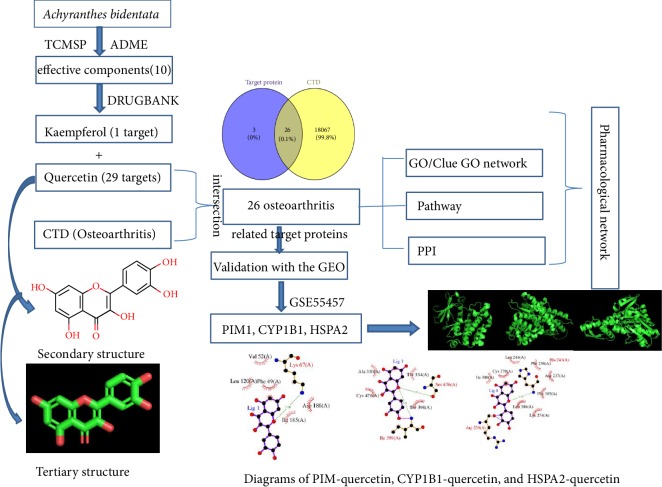
The overall design of this study and the main results.

As shown in Figure 1, the effective components of* Achyranthes bidentata* were firstly screened out from the TCMSP database with ADME property parameters. Then, proteins targeted by the effective components were predicted based on the TCMSP and DrugBank database. Keyword “osteoarthritis” was entered into the CTD database to search for the relevant genes. Based on the above results, there were 26 members in the intersection. Subsequently, the enrichment analysis and the interaction network of these target proteins were performed and constructed. After validation by GSE55457, PIM1, CY1B1, and HSPA2 were considered as the key proteins during the treatment of osteoarthritis by quercetin. Finally, the docking models of PIM-quercetin, CYP1B1-quercetin, and HSPA2-quercetin were predicted.

### 3.2. Effective Components of Achyranthes bidentata and the Target Proteins

**Figure 2:**
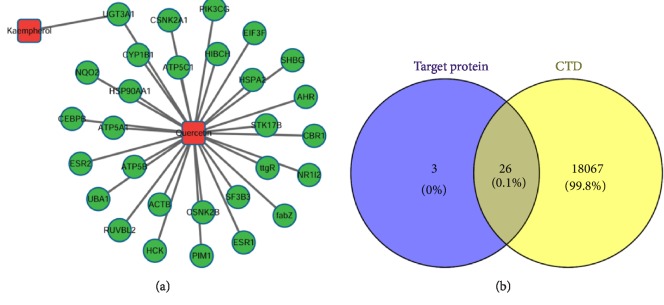
The network diagram of the relationship between effective components of* Achyranthes bidentata* and their target proteins (a). (b) Venn diagram of target protein of quercetin and osteoarthritis-related proteins based on the CTD database.

A total of 176 kinds of herbal ingredients were searched for in the TCMSP database with the key word “*Achyranthes bidentata*”. With the OB≥40% and DL≥0.2, 10 effective components of* Achyranthes bidentata *were obtained (Table 1). The above 10 components were searched for one by one in the DrugBank database. The results showed that there were two components with target records, namely, quercetin and kaempferol, with 29 targets and 1 and target, respectively. The network of quercetin and kaempferol with their target proteins was shown in Figure 2(a). There were 31 nodes (quercetin and kaempferol and their target proteins) and 30 relationship pairs in this network.

There were 26 proteins in the intersection of 29 targeted proteins of quercetin and the osteoarthritis-related proteins in the CTD database. The Venn diagram in Figure 2(b) and Table 2 listed the 26 osteoarthritis-related proteins.

### 3.3. Enrichment Analysis of 26 Targeted Proteins of Quercetin

**Figure 3:**
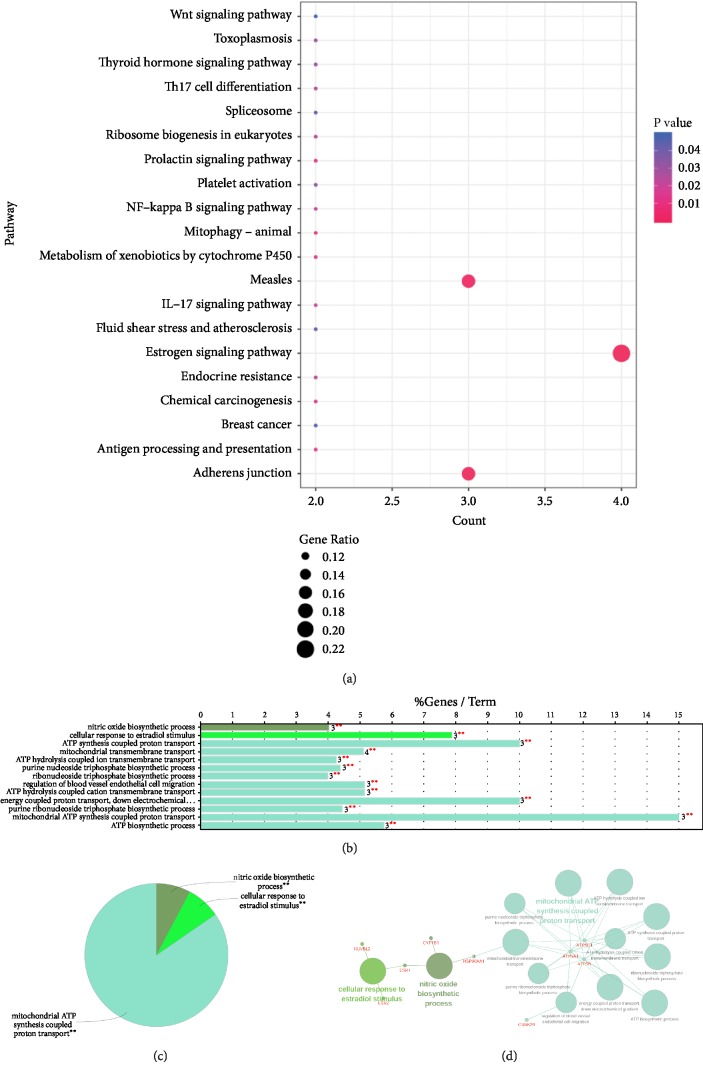
The enrichment analysis of 26 key target protein of quercetin. (a) The bubble map of enriched pathways. The vertical axis was the name of the pathway, the horizontal axis was the number of enriched genes, and the dot size was the ratio of the number of enriched genes to the total number of uploaded genes. The redder the dot color, the more significant the P values. (b), (c) Go function enrichment analysis. The vertical axis presented the names of the GO items; the abscissa represented the percentage of the enriched genes. (d) ClueGO Functional Network Diagram. The dots with red labels were target proteins and circles were the GO functions. The larger the P value, the larger the circle. The lines between two points represented the correlation between functions, and the larger the kappa coefficient, the thicker the line.

Enrichment analysis of 26 targeted proteins of quercetin was performed by the clusterProfiler package. As shown in Figure 3(a) the 26 proteins are mainly enriched in several pathways, such as Estrogen signaling pathway (ESR1/ESR2/HSP90AA1/HSPA2), Adherens junction (ACTB/CSNK2A1/CSNK2B), and Measles (CSNK2A1/CSNK2B/HSPA2). The GO function enrichment analysis showed that the targeted proteins are mainly related to 13 biological process items (Figure 3(b)). Based on the kappa value, the GO items were divided into three categories, nitric oxide biosynthetic process, cellular response to estradiol stimulus, and mitochondrial ATP synthesis coupled proton transport (Figure 3(c)).  Figure 3(d) showed the GO function network; we found that a total of 9 target proteins which belonged to quercetin were significantly enriched in the 13 GO items. The most important three Go items were mitochondrial ATP synthesis coupled proton transport (the matching target proteins were ATP5C1, ATP5A1, and ATP5B), cellular response to estradiol stimulus (the matching target proteins were ESR1, ESR2, and RUVBL2), and nitric oxide biosynthetic process (the matching target proteins were ESR1, CYPIB1, and HSP90AA1).

### 3.4. PPI Network Based on the Target Proteins of Quercetin

**Figure 4:**
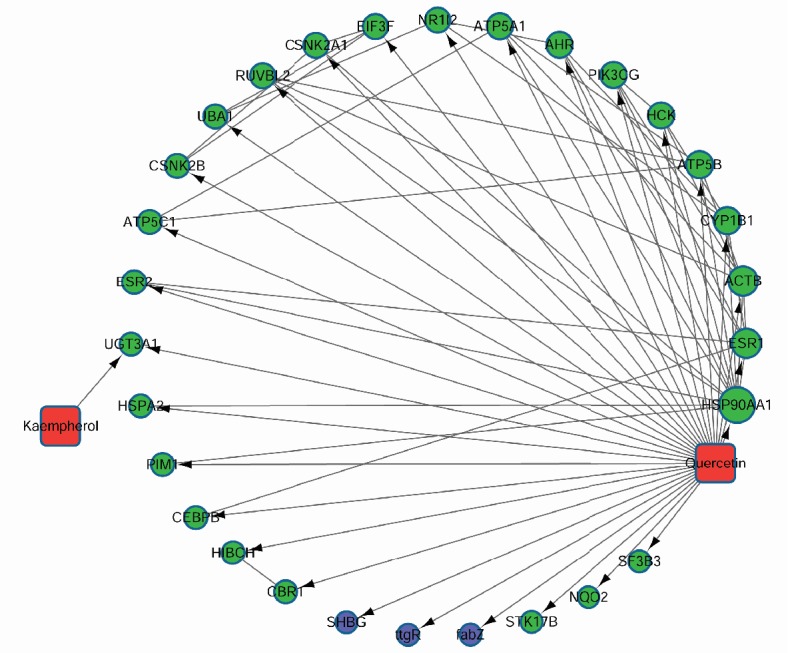
The PPI network of the target proteins of* quercetin*.

Figure 4 displayed the PPI network of the target proteins of* quercetin*. The connectivity in the network represented the correlation of two molecules. Based on the connectivity of proteins in the PPI network, the top 5 members were HSP90AA1 (degree was 13), ACTB (degree was 7), ESR1 (degree was 7), ATP5B (degree was 5), and CYP1B1 (degree was 5).

### 3.5. Pharmacological Network

**Figure 5:**
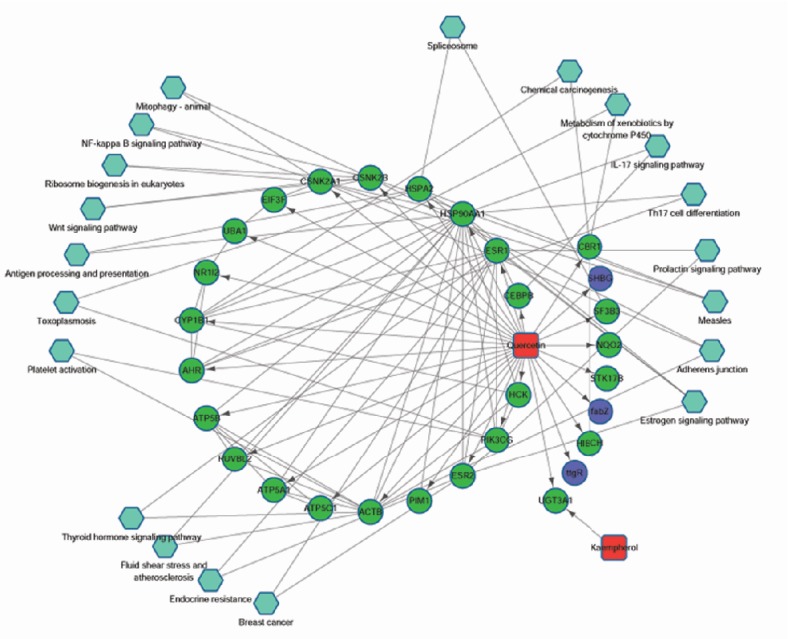
Pharmacology network based on the kaempferol, quercetin, their target proteins, and enriched pathways. The blue hexagons represented the pathways; the green dots were the target proteins.

Pharmacological network was constructed based on the effective components, target proteins, and pathways. As shown in Figure 5, there are 51 nodes in the network, with 113 relationship pairs, including 2 herbal component nodes, 29 target protein nodes, and 20 pathway nodes.

### 3.6. Verification of the Target Protein by GSE55457

A total of 557 DEGs between OA and control groups were screen out from the GSE55457. Compared with the target proteins of quercetin, there were three common members, protooncogene serine/threonine-protein kinase Pim-1 (PIM1), cytochrome P450 1B1 (CYP1B1), and heat shock-related 70 kDa protein 2 (HSPA2), which were considered as the key proteins during the treatment of osteoarthritis by quercetin.

### 3.7. Prediction of Key Target Protein and Quercetin Docking

**Figure 6:**
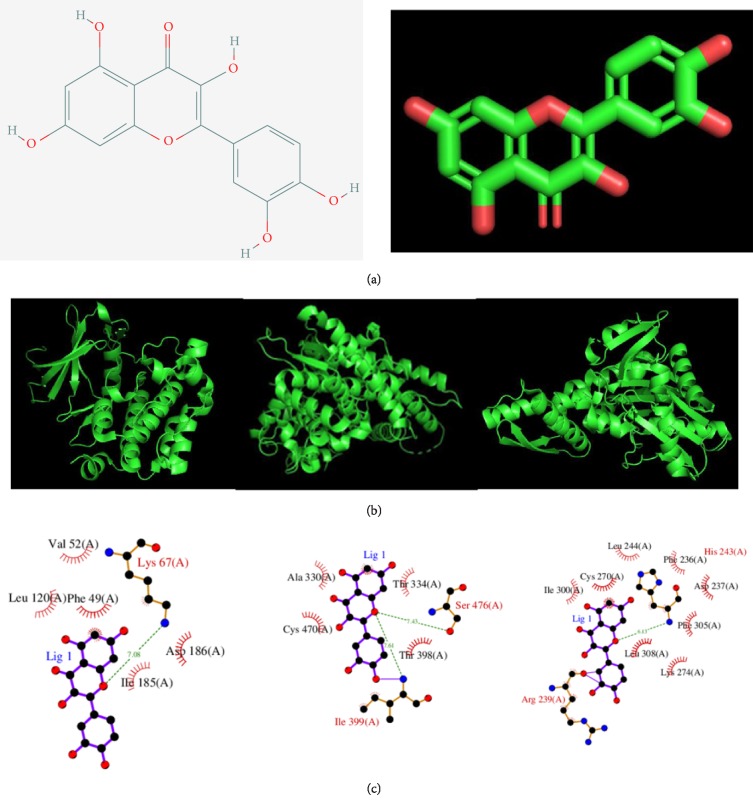
Structure predictions of quercetin, PIM-quercetin, CYP1B1-quercetin, and HSPA2-quercetin. (a) The secondary and tertiary structure of quercetin. (b) The 3D structures of PIM1 (left), CYP1B1 (middle), and HSPA2 (right). (c) The diagrams of protein-ligand interaction of PIM-quercetin (left), CYP1B1-quercetin (middle), and HSPA2-quercetin (right). The interactions were mediated by hydrogen bonding and hydrophobic contact. The dashed lines indicated hydrogen bonds; the arcs indicated hydrophobic contacts. Black dots represented carbon, red dots were oxygen, and blue dots were nitrogen. The black character labels were amino acids.

The secondary and tertiary structure of quercetin were downloaded from the DrugBank database and shown in Figure 6(a). Besides, the 3D structures of PIM1, CYP1B1, and HSPA2 were constructed and shown in Figure 6(b). After prediction of potential docking target of quercetin, the PBD IDs of PIM1, CYP1B1, and HSPA2 were 1XWS, 3PM0, and 4FSV, respectively. In addition, the corresponding docking scores (pKd/pKi) were 6.526, 6.968, and 6.334.  Figure 6(c) showed the diagram of protein-ligand interaction of PIM-quercetin, CYP1B1-quercetin, and HSPA2-quercetin.

## 4. Discussion

This study aimed to analyze the potential mechanism of treatment of osteoarthritis by* Achyranthes bidentata*. After comprehensive analysis of TCMSP, DrugBank, and CTD databases by the key words of “*Achyranthes bidentata”* and “osteoarthritis”. A total of 26 target proteins of quercetin related to the osteoarthritis were obtained, mainly enriched in the mitochondrial ATP synthesis coupled proton transport, cellular response to estradiol stimulus, and nitric oxide biosynthetic process. By validation of DEGs of GSE55457, three common proteins, PIM1, CYP1B1, and HSPA2, were considered as the key targeted proteins of the quercetin during the treatment of osteoarthritis by* Achyranthes bidentata*.

Quercetin is categorized as a flavonol, one of the six subclasses of flavonoid compounds [30]. Plenty studies have reported that quercetin showed many potential beneficial effects on human health, such as antioxidant activity and effects against cancer, cardiovascular disease, diabetes and diabetic complications, hypertension, immunity and infections, and arthritis [31]. Application of quercetin might reduce symptoms of osteoarthritis. Matsuno* et al*. reported that pain symptoms, daily activities, visual analogue scale, and the synovial fluid properties were significantly improved after application of quercetin glucoside for osteoarthritis patients [32]. Besides, quercetin could inhibit the activities of many kinases implicated in cancer cell biology, such as ABL1, Aurora-A, -B, -C, CLK1, FLT3, JAK3, and MET [33]. In our study, quercetin was the most important effective component during the treatment of osteoarthritis by* Achyranthes bidentata *based on searching several databases.

The normal nitric oxide biosynthetic process is important for human health. Increasing of nitric oxide production was detected in osteoarthritic joints indicating that nitric oxide was involved in the pathogenesis of osteoarthritis [34]. The nitric oxide production was regional differences in the knee meniscus in response to dynamic compression [35]. We found that the 26 target proteins of quercetin related to the osteoarthritis were mainly enriched in the nitric oxide biosynthetic process, indicating that nitric oxide related signal may play an important role in the treatment of osteoarthritis by* Achyranthes bidentata.*

PIM1 is a serine/threonine kinase encoded by the protooncogene Pim-1. PIM1 is mainly involved in cell cycle progression, apoptosis, and transcriptional activation, as well as more general signal transduction pathways [36]. Merkel* et al.* reported that PIM1 kinase had the potential as a target for cancer therapy [37]. Besides, many reports have shown that the activity of PIM1 could be inhibited by quercetin [38, 39]. In our study, Figure 5 showed that the quercetin regulated the PIM1, which was involved in the breast cancer. However, few literature works studied the correlation between PIM1 and osteoarthritis. CYP1B1 belongs to the cytochrome P450 superfamily of enzymes, which catalyze many reactions related to drug metabolism and synthesis of cholesterol, steroids, and other lipids [40]. We found that CYP1B1 was the target of quercetin, which was consistent with the previous reports. Choi* et al.* reported that quercetin acted as an antioxidant and downregulated CYP1B1 against 7,12-dimethylbenz(a)anthracene-induced oxidative stress in mice [41]. Quercetin showed strong and selective inhibition against CYP1B1, which required 2-3 double bonds on the C-ring [42]. HSPA2 is a 70-kilodalton heat shock protein, which plays a key role in regulating the recognition of sperm and egg [43]. Wang* et al. *reported that as the target of quercetin, HSPA2 might act as the ATP binding domain [44]. Although, there were few reports on the directed interaction of PIM1, CYP1B1, HSPA2, and osteoarthritis, the docking of PIM1-quercetin, CYP1B1-quercetin, and HSPA2-quercetin may play important roles during the treatment of osteoarthritis by* Achyranthes bidentata.* The expression levels and activities of PIM1, CYP1B1, and HSPA2 in the animal models and clinical samples have been arranged into our next research plan.

In conclusion, the docking of PIM1-quercetin, CYP1B1-quercetin, and HSPA2-quercetin may play important roles during the treatment of osteoarthritis by* Achyranthes bidentata*.

## Figures and Tables

**Table 1:** Effective components of* Achyranthes bidentata*.

Mol ID	Molecule Name	OB	DL
MOL001006	poriferasta-7,22E-dien-3beta-ol	42.98	0.76
MOL012537	Spinoside A	41.75	0.4
MOL012542	*β*-ecdysterone	44.23	0.82
MOL002776	Baicalin	40.12	0.75
MOL002897	epiberberine	43.09	0.78
MOL000422	kaempferol	41.88	0.24
MOL004355	Spinasterol	42.98	0.76
MOL000449	Stigmasterol	43.83	0.76
MOL000785	palmatine	64.6	0.65
MOL000098	quercetin	46.43	0.28

**Table 2:** The list of 26 osteoarthritis related proteins.

The intersection of 29 targeted proteins of quercetin and the osteoarthritis related proteins in the CTD database						
UGT3A1	ATP5A1	PIM1	ESR1	AHR	CSNK2A1	NR1I2
HCK	ATP5B	HIBCH	ESR2	CYP1B1	CSNK2B	RUVBL2
PIK3CG	ATP5C1	STK17B	NQO2	ACTB	EIF3F	UBA1
HSP90AA1	SF3B3	CEBPB	CBR1	HSPA2		

## Data Availability

The data used to support the findings of this study are available from the corresponding author upon request.
